# Severe Colchicine Toxicity After Ingestion of a Dose Below Traditionally Reported Lethal Levels: A Case Report

**DOI:** 10.7759/cureus.116034

**Published:** 2026-09-10

**Authors:** Seena Sugathan, Vishalini Ammampalayam Venkatachalam, Aisha Khan, Aya Twabie

**Affiliations:** 1 Acute Medicine, North Manchester General Hospital, Manchester University NHS Foundation Trust, Manchester, GBR

**Keywords:** colchicine, colchicine overdose, colchicine poisoning, drug-induced bone marrow suppression, drug overdose and poisoning, multiorgan system failure

## Abstract

Colchicine is a widely utilised drug for the management of conditions such as gout, pericarditis and familial Mediterranean fever. It has a narrow therapeutic index, with a significant risk of toxicity in overdose leading to life-threatening consequences. We present a case of severe colchicine toxicity following ingestion of 20 mg (0.26 mg/kg), resulting in multi-organ failure requiring intensive care management. Rapid deterioration with hyperthermia, electrolyte derangement, rhabdomyolysis, coagulopathy and significant bone marrow suppression necessitated IV antibiotics, platelet transfusion and granulocyte colony-stimulating factor (GCSF) and subsequent admission to critical care for vasopressors and ventilatory support. The patient eventually improved with resolution of haematological and renal parameters, was reviewed by the mental health team and discharged with full functional recovery. This case underscores the importance of early recognition, aggressive supportive care, and multidisciplinary management of patients presenting with colchicine overdose, in order to achieve a favourable outcome. The unpredictable relationship between colchicine dose and clinical outcome highlights the need for close monitoring even following relatively low-dose ingestion. Increased clinician awareness of colchicine toxicity and its delayed manifestations is essential for timely intervention in such critical cases.

## Introduction

Colchicine is a naturally occurring alkaloid widely used in the management of acute gout, for prophylaxis during urate-lowering therapy, and in conditions such as familial Mediterranean fever, mainly due to its potent anti-inflammatory action [1]. Despite its well-established therapeutic effects, colchicine has a very narrow safety margin, and even slight overdoses can lead to severe toxicity, resulting in significant morbidity and mortality [2]. The risk of adverse outcomes is notably higher in patients with pre-existing renal or liver derangement, cardiac comorbidities, gastrointestinal disease, and in the elderly population. Clinical manifestations may develop within a few hours of ingestion, but in some instances, they can be delayed for several days, creating diagnostic challenges [2].

As an anti-mitotic agent, colchicine exerts its anti-inflammatory action by binding to tubulin, which inhibits microtubule formation and disrupts essential intracellular processes [1,2]. This mechanism also contributes to its potential toxicity, as it impairs cellular division and neutrophil function. Colchicine and its metabolites also undergo significant first-pass metabolism, leading to reduced elimination from the body and further increasing its toxic side effects [1,3].

Although reported cases of colchicine overdose are rare, severe poisoning carries a high mortality rate, particularly when diagnosis or treatment is delayed. The relationship between the ingested dose and clinical outcome is highly variable, and there is no clearly defined dose that reliably distinguishes toxic from lethal exposure. Fatalities have been reported following ingestions as low as 7-26 mg, while survival has also been reported following substantially larger ingestions [2]. The risk of life-threatening side effects is increased by concomitant use of drugs such as P-glycoprotein inhibitors (e.g. cyclosporine, ranolazine) and CYP3A4 inhibitors (e.g. itraconazole, clarithromycin, ketoconazole, and some protease inhibitors) [3].

We describe a case of acute colchicine toxicity that resulted in multiorgan dysfunction, bone marrow suppression, rhabdomyolysis, and sepsis. Despite an ingestion of 20 mg (0.26 mg/kg), the patient developed severe and prolonged multiorgan toxicity requiring intensive care management and subsequently achieved full functional recovery. This case underscores the importance of maintaining clinical vigilance and recognising the potentially delayed and life-threatening nature of colchicine poisoning.

## Case presentation

A 53-year-old male patient with a background of gout presented to the Emergency Department with abdominal pain, vomiting and diarrhoea following intentional ingestion of 40 tablets of 0.5 mg colchicine (total consumption: 20 mg; 0.26 mg/kg) five hours earlier. On clinical review, he was found to be tachycardic but otherwise haemodynamically stable. His laboratory investigations revealed erythrocytosis, leucocytosis, neutrophilia, lymphocytosis, basophilia and increased reticulocytes, with a normal monocyte and eosinophil count (Table 1). Approximately five hours after ingestion, following discussion with the TOXBASE consultant, whole-bowel irrigation was commenced via a nasogastric tube using Klean-Prep to reduce ongoing gastrointestinal absorption of residual colchicine. The patient tolerated approximately 3,000 mL (the recommended dose is 2,000 mL every two hours for six hours) before the procedure was discontinued due to intolerance; activated charcoal was not administered.

**Table 1:** Laboratory Investigations from admission till day 12 WBC: white blood cell count; Hb: haemoglobin; eGFR: estimated glomerular filtration rate; ALT: alanine aminotransferase; ALP: alkaline phosphatase; CRP: C-reactive protein; CK: creatine kinase; BNP: B-type natriuretic peptide; Mg: magnesium; PO_4_: phosphate; PCT: procalcitonin; INR: international normalised ratio; PT: prothrombin time; aPTT: activated partial thromboplastin time

Parameter	Normal Range	0 Hour	12 Hours	Day 2	Day 3	Day 4	Day 5	Day 6	Day 7	Day 8	Day 9	Day 10	Day 11	Day 12
WBC	4-11 x 10^9^/L	23.3	22	17.7	6.3	0.4	0.3	0.3	0.9	2.4	12.8	25.7	27.6	26.6
Hb	130-180 g/L	200	180	175	148	134	123	116	107	100	108	103	98	90
Neutrophil	1.80-7.50 x 10^9^/L	18.13	16.91	14.03	5.14	0.2	0.04	0.03	0.82	8.71	18.48	19.14	20.66	13.35
Lymphocyte	1.00-4.00 x 10^9^/L	4.2	4.43	3.31	1.10	0.62	0.14	0.16	0.67	2.24	4.88	6.24	4.36	2.99
Basophils	0.00-0.10 x 10^9^/L	0.35	0.33	0.18	0.02	0.01	0	0	0.02	0.02	0.03	0.18	0.11	0.05
Reticulocytes	20-80 x 10^9^/L	119	-	59	22	14	11	9	9	16	20	41	-	-
Platelets	150-400 x 10^9^/L	320	198	109	52	9	11	12	13	53	39	41	68	89
Monocytes	0.2-0.8 x 10^9^/L	0.48	0.21	0.08	0.02	0.01	0.03	0.1	0.86	1.73	2.16	1.94	1.4	0.84
Urea	2.5-7.8 mmol/L	8.3	13.4	19.5	15.8	11.2	9.6	10.5	10	10.2	7.7	8.7	10.4	10.7
Creatinine	59-104 μmol/L	184	230	245	110	93	84	76	74	80	77	88	92	84
eGFR	mL/min/1.73m^2^	35	27	33	66	81	86	>90	>90	>90	>90	86	82	>90
ALT	1-50 IU/L	-	-	-	-	187	296	346	409	291	254	251	231	-
ALP	30-130 IU/L	217	412	597	528	375	100	90	111	125	180	178	176	164
T-bilirubin	0-21 μmol/L	5	5	6	8	27	53	93	87	57	39	23	17	-
CRP	0-5 mg/L	28	131	155	110	110	250	-	146	168	153	146	128	42
Lactate	0.3-2 mmol/L	5.4	3.1	3.1	2.6	2.7	-	-	-	-	-	-	1.7	1.7
D-dimer	0-500 ng/mL	-	-	35,000	-	-	-	-	-	-	-	-	-	-
Lipase	13-60 U/L	257	-	-	-	-	-	-	-	-	-	-	-	-
CK	40-320 IU/L	1071	1632	2743	8858	14,774	51,022	-	35,319	13,448	14,595	18,469	9702	5422
BNP	0-400 ng/L	-	-	-	-	-	2478	-	2619	-	458	-	271	-
Mg	0.70-1.00 mmol/L	0.73	-	0.75	0.95	-	1.13	1.14	1.18	1.11	1.05	0.95	0.78	-
Adjusted calcium	2.20-2.60 mmol/L	1.92	-	1.88	2	-	1.93	1.65	1.64	1.85	2.11	2.3	2.34	2.22
PO_4_	0.80-1.50 mmol/L	1.42	-	1.58	0.82	-	0.39	0.39	0.58	0.43	0.76	0.88	0.86	-
PCT	-	-	-	-	0.92	-	5.4	3.39	3	2.37	1.89	2.63	2.52	1.93
Fibrinogen	1.7-4.2 g/L	-	-	2.2	-	3.6	-	6.8	-	-	-	-	-	-
INR	1-5 ratio	-	1.95	2.11	1.16	1.17	1.38	1.18	1.24	1.21	1.12	1.15	1.14	-
PT	9.9-11.8 seconds	-	16.5	16.6	11.9	12	-	12.1	12.6	12.4	11.5	11.8	11.7	11
aPTT	21-30 seconds	-	27.7	38.7	28.8	27.9	-	22.5	23.4	21.6	20.4	21.2	22.2	20.7
Troponin T	0-14 ng/L	9	-	-	-	-	186	152	129	-	-	177	185	-

Blood tests also showed acute kidney injury (AKI) and electrolyte derangement, particularly hypocalcaemia (Table 1), along with high levels of CRP, alkaline phosphatase (ALP) and lactate. Creatine kinase was increased to 1071 (Figure 1), and lipase was 257. Blood gases were unremarkable. His coagulation profile was deranged, with an elevated prothrombin time (PT) and normal activated partial thromboplastin time (APTT) (Figure 2). On the second day of admission, his renal, haematological and liver parameters continued to decline, along with hyperthermia, worsening coagulopathy and rhabdomyolysis, suggesting multiorgan failure (Table 1). A CT pulmonary angiography was performed using an Aquilion PRIME 160-slice CT scanner (Canon Medical Systems Corporation, Otawara, Tochigi, Japan) due to respiratory compromise, which ruled out pulmonary embolism and revealed underlying lung consolidation warranting treatment for aspiration pneumonia with antibiotics.

**Figure 1:**
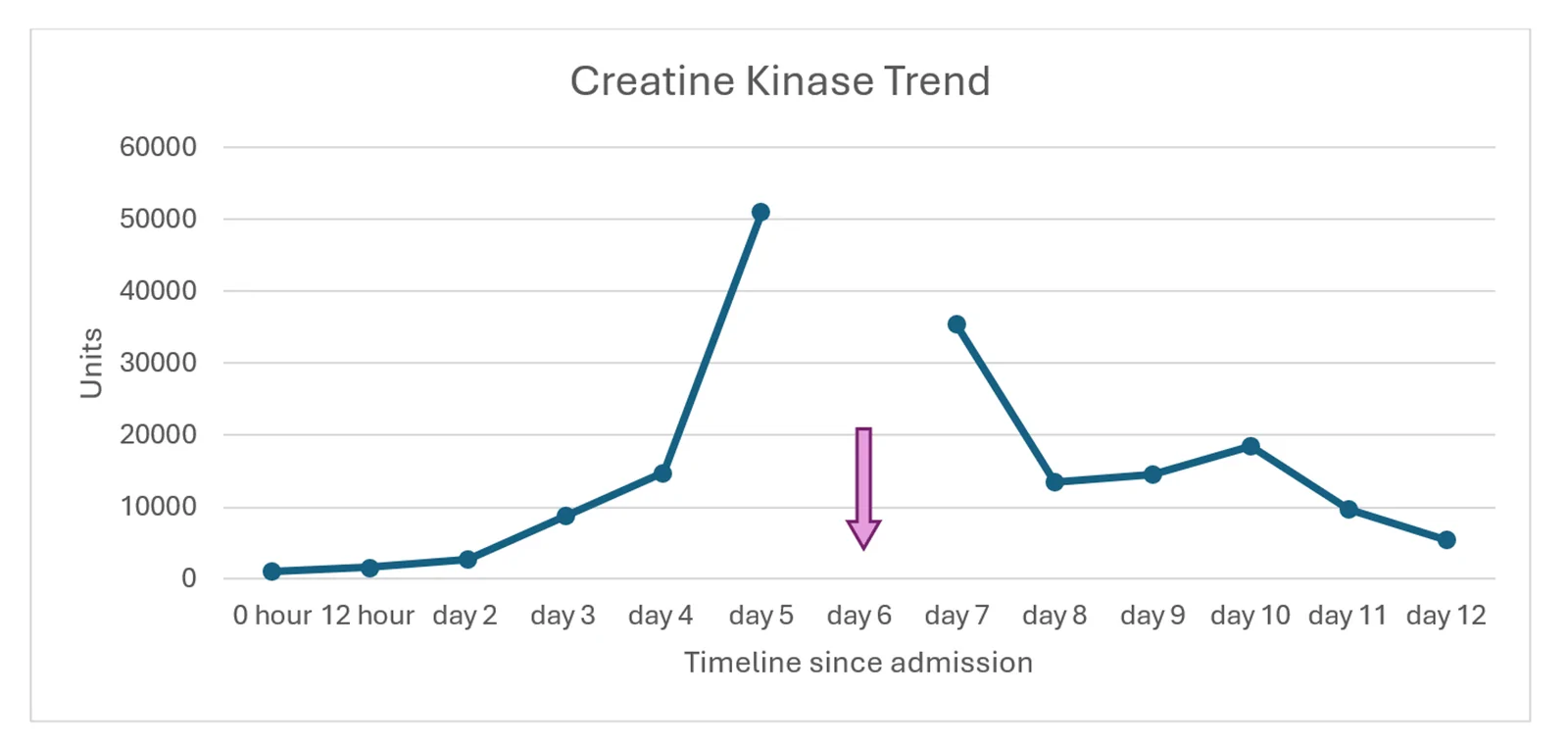
The trend of creatine kinase since the day of admission The graph demonstrates the creatine kinase trend in relation to the severity and progression of muscle injury associated with rhabdomyolysis. The violet arrow indicates the day on which creatine kinase monitoring was not performed.

**Figure 2:**
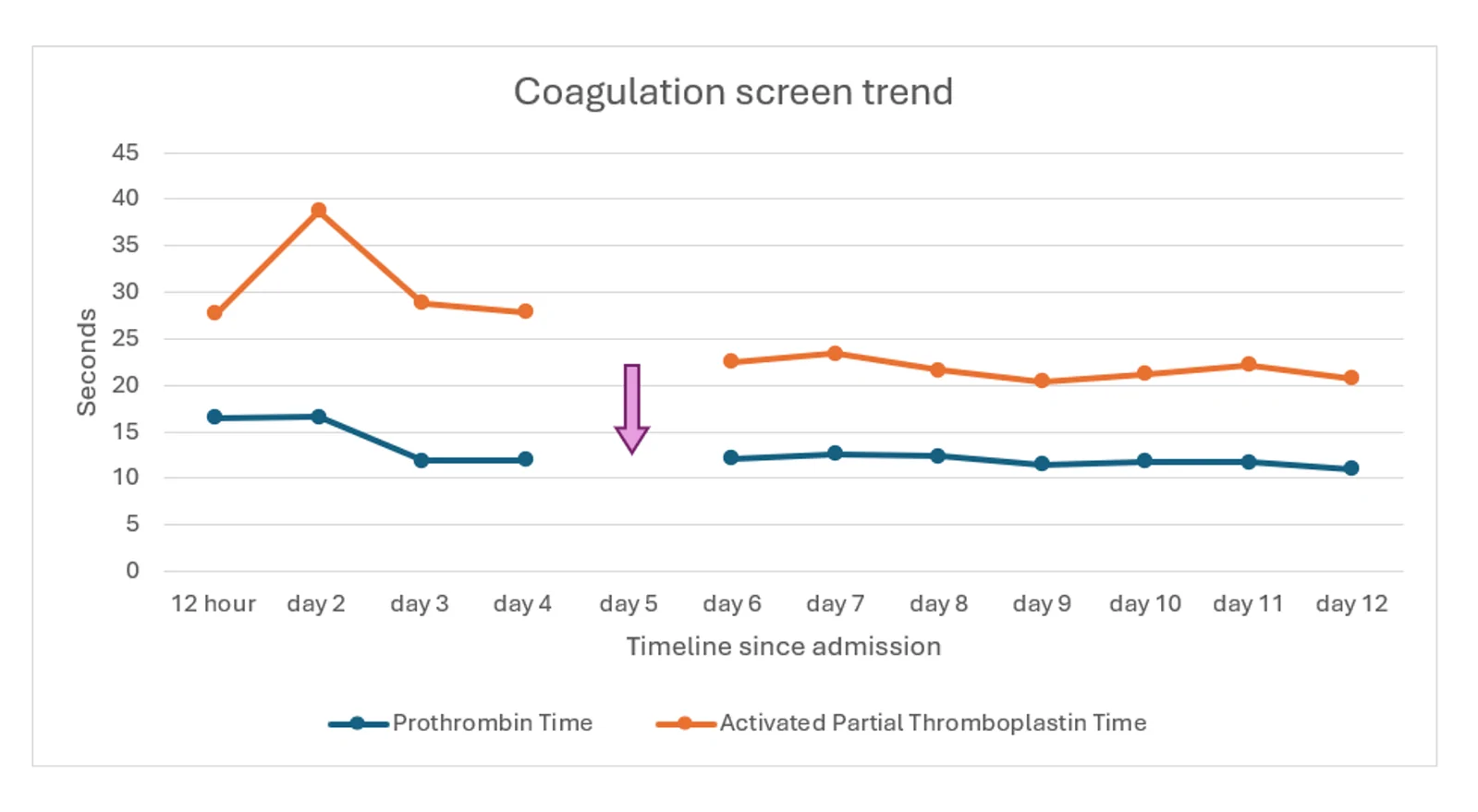
The coagulation screen trend since admission The graph represents the rise and fall of the coagulation screen (prothrombin time and activated partial thromboplastin time). The violet arrow depicts the days on which the coagulation screen was not monitored.

On the fourth day, the patient developed signs of spontaneous bleeding (including bleeding gums, conjunctival haemorrhage and spontaneous bruising in lower limbs) associated with profound thrombocytopenia, leukopenia and dropping haemoglobin secondary to bone marrow suppression (Table 1 and Figures 3-4). He was given platelet transfusion along with granulocyte colony-stimulating factor (G-CSF) injection following haematology consultation, and was treated for neutropenic sepsis (Figure 5).

**Figure 3:**
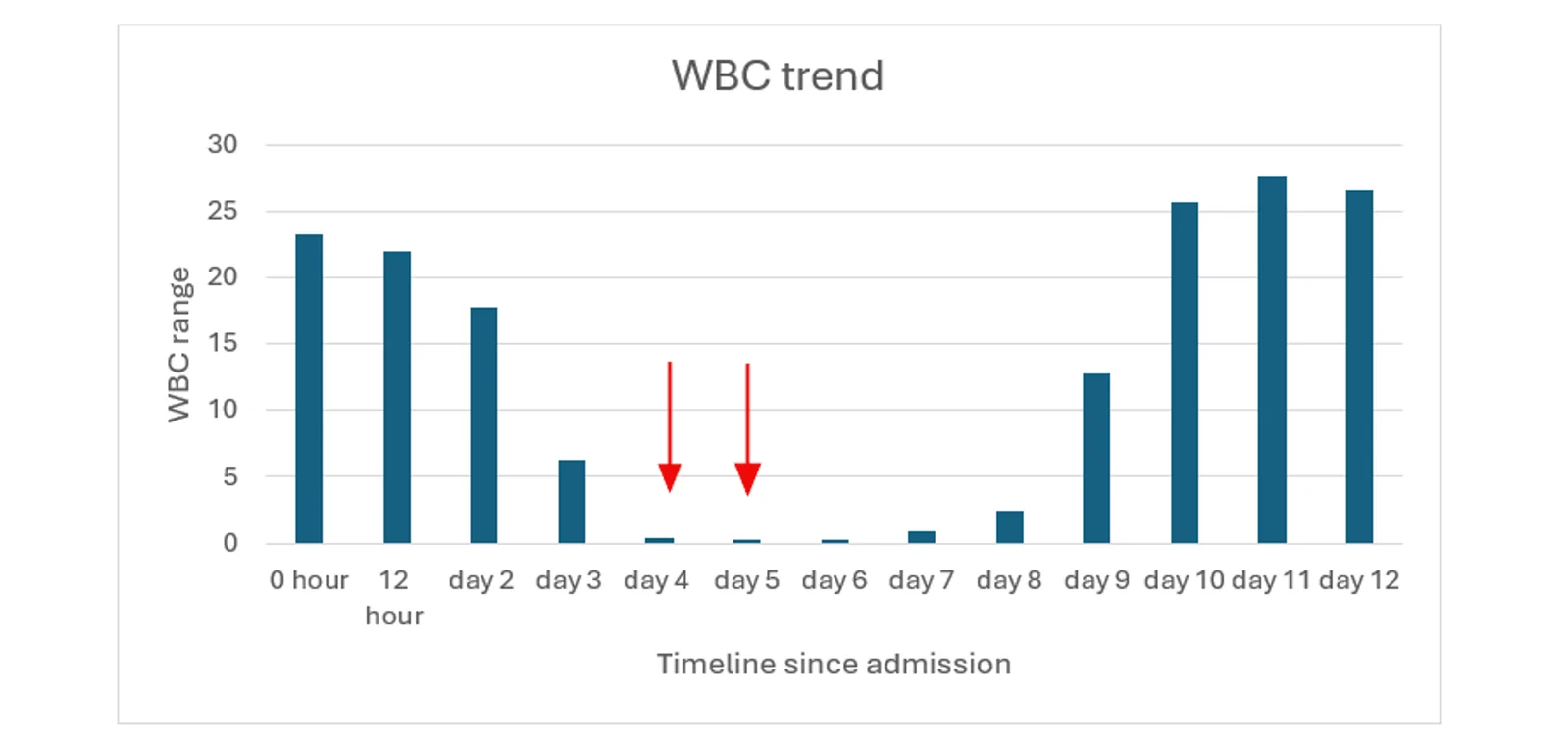
The trend of white blood cell (WBC) since admission to day 12 The red arrows indicate the days on which granulocyte colony-stimulating factor (G-CSF) injections were administered. The graph illustrates the development and subsequent recovery of leukopenia and its temporal relationship with G-CSF administration.

**Figure 4:**
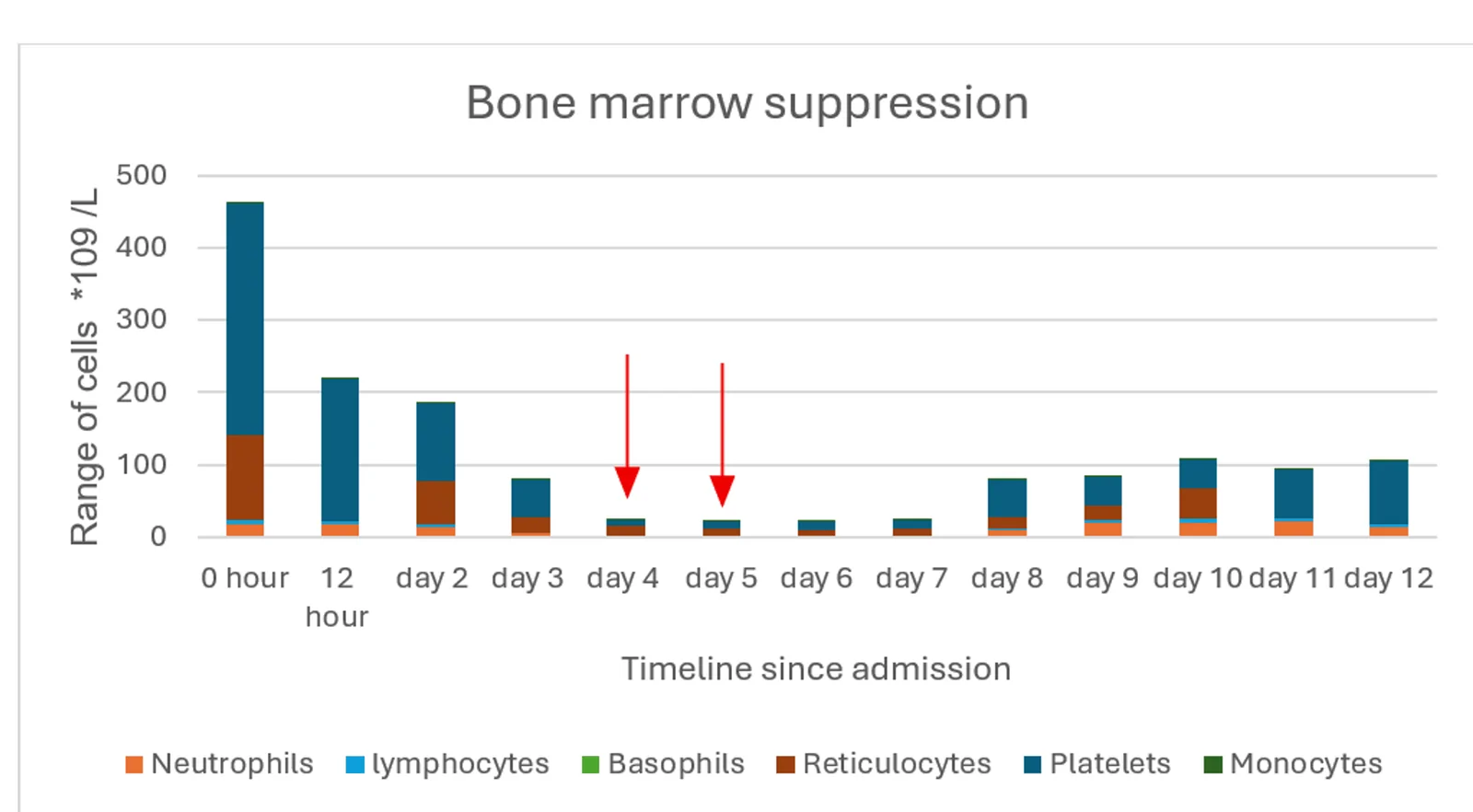
The trend of the various components of white blood cells since admission The red arrows indicate the day of administration of granulocyte colony-stimulating factor (G-CSF) injections, and the trends in the white blood cell differential demonstrate the development of cytopenia and subsequent improvement following G-CSF administration.

**Figure 5:**
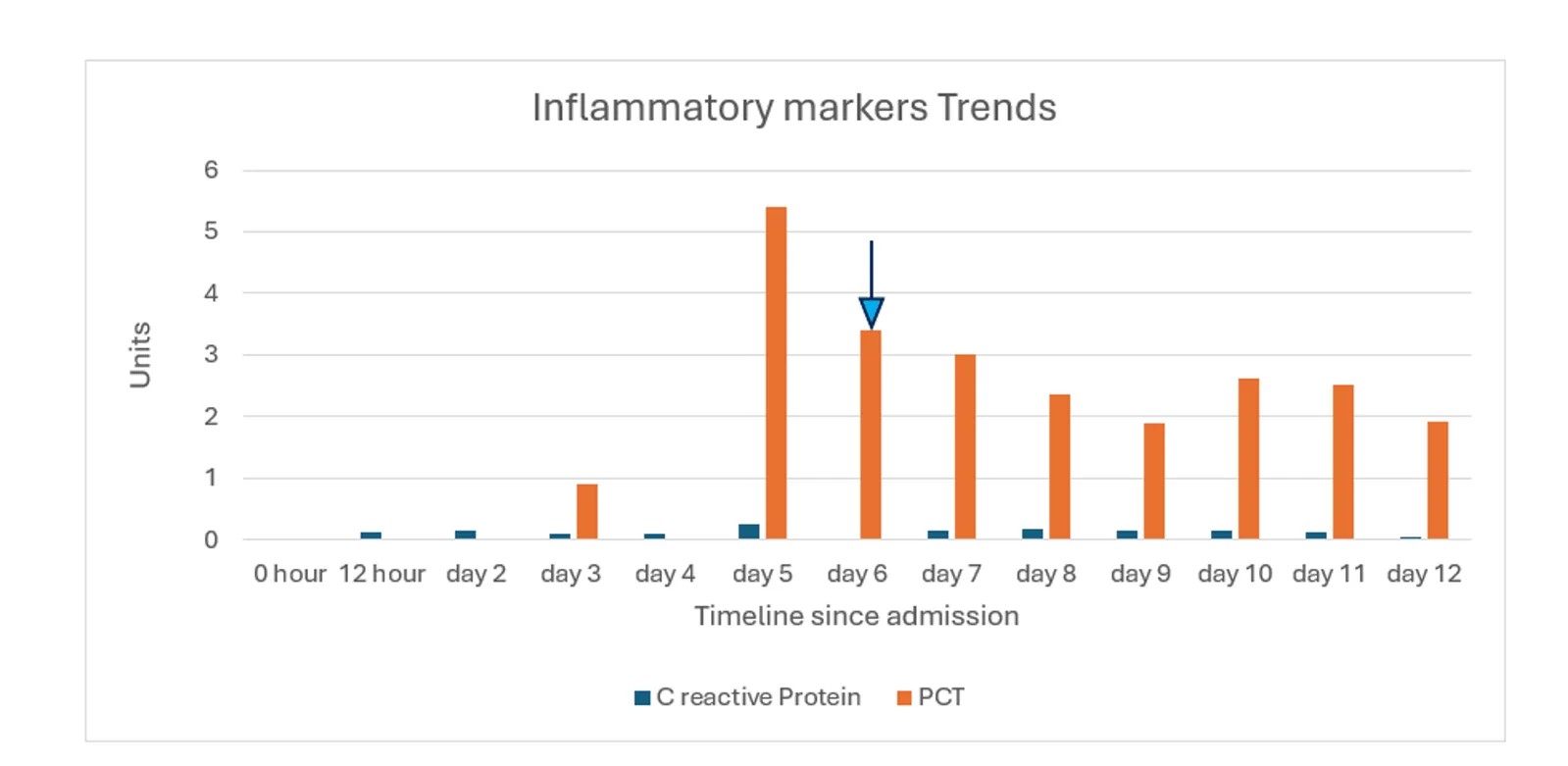
The trend of inflammatory markers since the day of the admission The graph represents the C-reactive protein (CRP) and procalcitonin (PCT) levels during the admission. It depicts the relationship between the CRP/PCT trends, neutropenic sepsis, clinical deterioration, and ICU admission on day 6. The blue arrow depicts admission to the ICU.

Despite early intervention, thrombocytopenia and leukopenia worsened along with increasing ventilatory requirement and delirium, necessitating escalation to the intensive care unit (ICU) for intubation and mechanical ventilation on the sixth day of admission. Derangement of electrolytes, particularly calcium and phosphate, was corrected with intravenous replacement (Figure 6). Bronchoalveolar lavage (BAL) sampling grew *Stenotrophomonas*, and antibiotics were escalated to co-trimoxazole and meropenem as per microbiology guidance.

**Figure 6:**
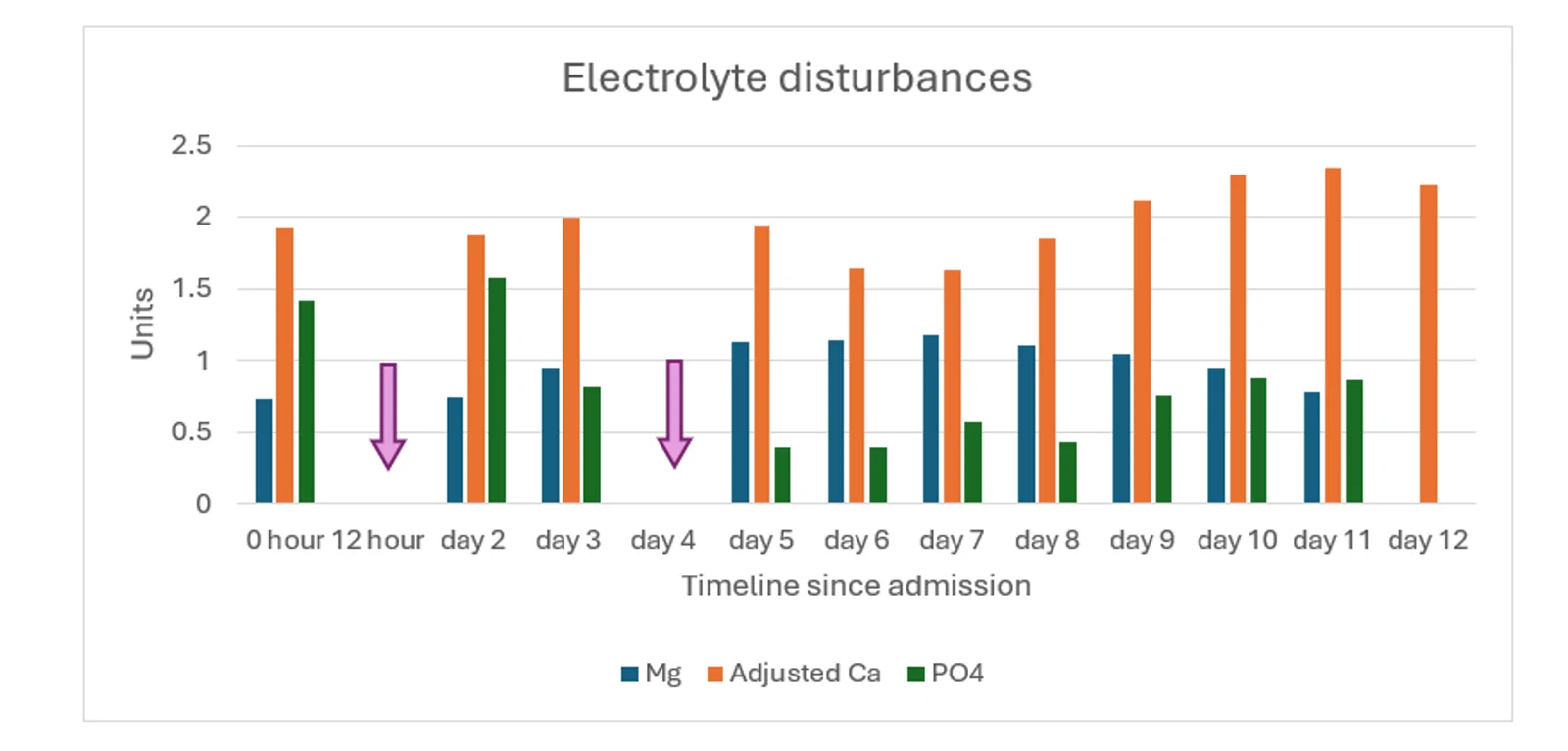
The trend of significant electrolyte disturbances in serum The graph represents the significant serum electrolyte derangements noted during the admission. The violet arrows depict the day with no serum monitoring, during which blood gases were used to monitor and correct the electrolyte derangements. Mg: magnesium; adjusted Ca: adjusted calcium; PO_4_: phosphate

The patient's oxygen requirement improved over the next few days, allowing successful extubation back to high-flow oxygen on day 19. Haematological and renal parameters improved significantly, with gradual resolution of AKI and bone marrow suppression. Following a ward stepdown, he transitioned from enteral to oral feeding, with resolution of delirium and near-resolution of laboratory parameters except mild liver derangement. He was reviewed by the mental health team with a plan for cognitive behavioural therapy (CBT), and was eventually discharged on day 27 with full functional recovery. The chronological progression of clinical manifestations and laboratory abnormalities is presented in Table 1 and Figures 1-7, highlighting the warning signs and subsequent progression to multiorgan toxicity.

**Figure 7:**
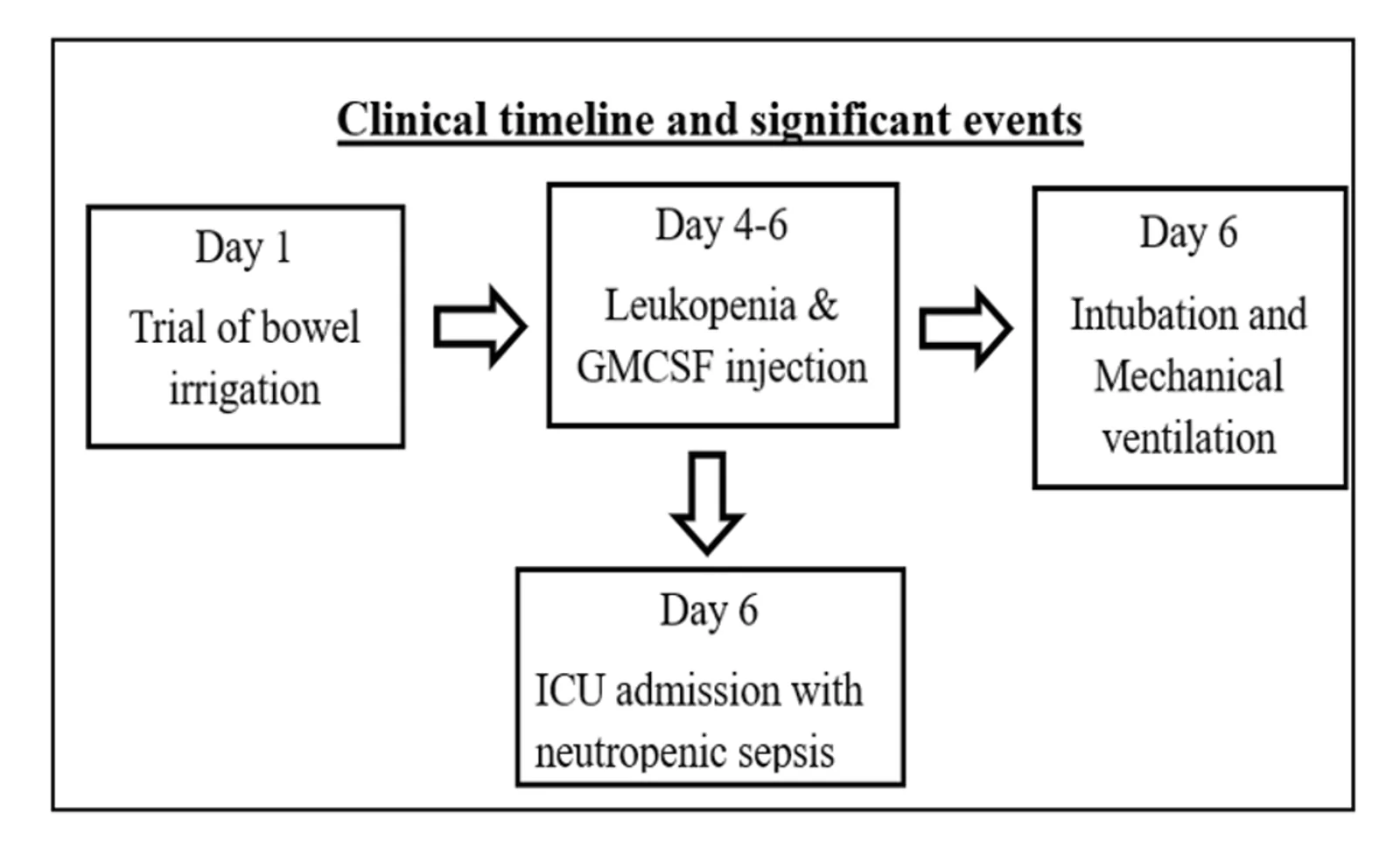
Key events and treatments during the inpatient stay The figure depicts the key clinical events, laboratory findings, and treatments administered during the inpatient stay. GMCSF: granulocyte-macrophage colony-stimulating factor

## Discussion

Although colchicine toxicity has historically been linked to large intentional ingestions, growing evidence indicates that severe toxicity can also occur following relatively low doses. The relationship between colchicine dose and clinical outcome is highly variable, and there is no clearly defined threshold below which severe or fatal toxicity can be excluded. In this case, the patient developed severe multiorgan toxicity following ingestion of 20 mg (0.26 mg/kg) [4]. Previous reports illustrate this variability. Finkelstein et al. reported fatal colchicine ingestions as low as 7-26 mg, while survival has also been reported following substantially larger ingestions of 60 and 71 mg [1]. Conversely, survivors have been reported following doses comparable to that in our patient. In a paediatric case series, patients recovered following reported ingestions of 0.11-0.6 mg/kg, while fatal cases occurred at 0.5-0.84 mg/kg and were associated with disseminated intravascular coagulation, cardiovascular shock and multiorgan failure. These reports support the view that colchicine toxicity is not solely dose-dependent. Instead, its severity is shaped by patient-specific variables including renal or hepatic impairment, age, comorbid conditions, genetic differences in drug metabolism, interacting medications, and delays in seeking medical care [5].

Colchicine exerts its toxic effects through potent inhibition of microtubule polymerisation. By binding to tubulin and preventing microtubule assembly, it disrupts mitosis, intracellular trafficking, and numerous essential cellular processes [6]. Tissues with rapid cell turnover, especially the gastrointestinal epithelium, bone marrow, and hair follicles, are therefore disproportionately affected [7]. Clinically, colchicine toxicity typically progresses through three phases: an early gastrointestinal phase marked by nausea, vomiting, diarrhoea, and abdominal pain; an intermediate phase characterised by multiorgan dysfunction, including hepatic injury, AKI, metabolic derangements, and bone marrow suppression; and, in severe cases, a late recovery phase associated with rebound leucocytosis and alopecia [8]. The patient’s course closely followed these expected stages despite the absence of a large overdose, underscoring the unpredictability of individual susceptibility.

Pharmacokinetic factors further complicate colchicine toxicity. Colchicine is metabolised predominantly by CYP3A4 and is a substrate of the P-glycoprotein (ABCB1) efflux transporter. Concomitant administration of potent CYP3A4 or P-glycoprotein inhibitors - including clarithromycin, ketoconazole, cyclosporine, verapamil, and certain protease inhibitors - can markedly increase systemic colchicine exposure and precipitate severe or fatal toxicity, even when colchicine is administered at therapeutic doses [1,9-11]. In the present case, no potentially interacting medications were identified, and the patient had normal renal and hepatic function prior to the event. Thus, no conventional pharmacokinetic risk factors for increased colchicine exposure were apparent, making the severity of toxicity following the reported 20 mg (0.26 mg/kg) ingestion particularly noteworthy.

Several reports document severe organ dysfunction, survival or fatal outcomes following colchicine ingestion in the range of 0.2-0.5 mg/kg, further demonstrating that toxicity does not correlate reliably with the amount ingested [10]. In our patient, the ingested dose of 0.26 mg/kg falls within a range in which both severe toxicity with survival and fatal outcomes have previously been reported. This variability highlights the limitations of relying exclusively on estimated dose when determining risk, disposition, or monitoring needs. An added challenge is the potential for delayed clinical deterioration. Even when early symptoms appear mild, progressive systemic toxicity may develop over 24-72 hours, underscoring the need for prolonged monitoring and repeated clinical evaluation.

Management remains primarily supportive. Early administration of activated charcoal may reduce systemic absorption when provided promptly, but its benefit decreases rapidly due to colchicine’s swift uptake [7]. Ongoing treatment focuses on hemodynamic stabilisation, management of metabolic abnormalities, respiratory support, and careful monitoring for hematologic suppression. Although colchicine-specific Fab antibody fragments have demonstrated rapid reversal of toxicity and improved outcomes in experimental studies and isolated clinical cases of severe colchicine poisoning, their use remains investigational because no commercially available formulation is available for routine clinical practice, limiting widespread adoption and further clinical evaluation [7,12]. Consequently, early recognition and aggressive supportive care are paramount.

## Conclusions

This case highlights the potential for severe colchicine toxicity following relatively low doses, despite the absence of identifiable renal or hepatic dysfunction or relevant drug interactions, contributing to the evidence that the relationship between ingested dose and clinical severity is highly variable. Clinicians should maintain a high index of suspicion, recognise early gastrointestinal symptoms as potential indicators of evolving systemic toxicity, and consider prolonged hospital monitoring even when the initial presentation appears mild. In the absence of an established specific antidote, vigilant supportive management and close clinical and laboratory monitoring remain essential for optimising outcomes in colchicine poisoning.
